# Influence of Enamel Exposure to Acidic Drink on Shear Bond Strength of Different Fissure Sealants

**DOI:** 10.3390/bioengineering9010020

**Published:** 2022-01-08

**Authors:** Riccardo Beltrami, Marco Colombo, Andrea Cavada, Sofia Panizzi, Claudio Poggio, Andrea Scribante

**Affiliations:** Section of Dentistry, Department of Clinical, Surgical, Diagnostic and Paediatric Sciences, University of Pavia, 27100 Pavia, Italy; marco.colombo@unipv.it (M.C.); andrea.cavada@icloud.com (A.C.); sofia.panizzi01@universitadipavia.it (S.P.); claudio.poggio@unipv.it (C.P.); andrea.scribante@unipv.it (A.S.)

**Keywords:** enamel erosion, fissure sealants, pretreatment, shear bond strength, soft drinks

## Abstract

In the present study, we evaluated the influence of bovine enamel exposure to acidic drinks (Coca-Cola, Coca-Cola Company, Milano, Italy, pH = 2.37) on shear bond strength of three sealants (Fissurit; Grandio Seal and Admira Fusion—Voco Gmbh, Cuxhaven, Germany). For each sealant, two adhesive techniques were tested to investigate the impact of the adhesive application on shear bond strength of sealants after immersion in acidic drink and in the control: Group 1—Control: enamel surface was not in contact with acid drinks, acid etching application and self-adhesive technique for fissure sealant; Group 2—enamel surface was not in contact with acid drinks, acid etching, and adhesive applications, an etch-and-rinse technique for fissure sealant; Group 3—enamel surface was immersed in acid drink, acid etching application and self-adhesive technique for fissure sealant; Group 4—enamel surface was immersed in acid drink, acid etching, and adhesive applications, an etch-and-rinse technique for fissure sealant. For each specimen, the sealant composite resin was applied to the enamel surface and tested with a universal testing machine. Shear bond strength was measured in MPa and with an optical microscope to determine failure modes, quantified with adhesive remnant index (ARI). Enamel acidification variably influenced bond strength values of the different sealants. When no enamel pretreatment was applied, no significant differences were found among the sealants (*p* > 0.05). However, the mere application of acid etching without adhesive procedures resulted in lower bond strength (*p* < 0.001). The acid pretreatment affected significantly the bond strength of all sealants tested (*p* < 0.001), but no significant differences were recorded between the subgroups.

## 1. Introduction

Fissure sealants are proved to be effective in the prevention of occlusal caries in molars and premolars [1,2,3,4]. Sealants create a physical barrier between the oral environment and pits and fissures of enamel where cariopathogenic dental biofilm is located [5,6,7]. In fact, the possibility to adhere to enamel tissue is the main prerequisite for the formation of the biofilm [8,9]. Smooth surfaces created with the application of fissure sealants should minimize susceptibility to adhesion for oral bacterial [10]. The success of this treatment is demanded by the bond strength of the sealants. The incomplete or low adhesion of the sealant to the enamel surface could create infiltration, secondary caries underneath the sealant, and detachment of the sealant [11].

In recent years the scientific and technological progress has brought to investigate new mini-invasive imaging methods for the diagnosis of initial caries and enamel infractions. For example, high-frequency ultrasound could become part of the daily routine because it provides precise information about the presence/absence of caries and the dimension [12]. However till now that these technologies are not widely applicable, the best treatment to prevent enamel from initial caries remains the fissure sealing [13,14].

Nowadays the current commercial type of sealant used is a resin-based nano-hybrid light-curing material. These types of materials require a modern system of adhesion, besides some of these, are now considered self-adhesive sealants. In literature, many authors studied the bond strength of self-adhesive resin composites to enamel and all of them showed unclear results. [6,7,8,9]. The etch-and-rinse technique remains the gold standard for adhesion of a resin-based material to untreated enamel [10,15,16].

However, even in young patients is hard to find untreated enamel for a lot of reasons [17,18,19,20,21,22,23]. Frequently young patients’ teeth are affected by caries, erosion and congenital anomalies and the use of a self-adhesive fissure sealant to prevent new caries is hard to achieve due to the high percentage of bonding failure [24,25]. Several studies investigated the impact of enamel erosion on the bond strength of restorative materials and it is clear that the etch-and-rinse technique minimizes the influence of an unhealthy substrate for adhesion [26]. In vitro studies could replicate the clinical conditions of an eroded enamel by immersion of specimens in acidic drinks for a prolonged time [27,28,29].

This study aims to compare the shear bond strength of different fissure sealants to untreated enamel versus eroded enamel, both when the self-adhesive technique is used both with etch-and-rinse. The tested hypothesis is that fissure sealants provide an acceptable shear bond strength when used on eroded enamel with a self-adhesive technique.

## 2. Materials and Methods

### 2.1. Preparation of Enamel Surface

Two hundred and forty bovine permanent incisors freshly extracted and stored in a solution of 0.1% (*wt*/*vol*) thymol were used as a substitute for human teeth [30,31]. A criterion for tooth selection included intact buccal enamel with no cracks caused by extraction. The teeth were cleaned of soft tissue and embedded in self-curing, fast-setting acrylic resin (Rapid Repair, DeguDent GmbH, Hanau, Germany). Specially fabricated cuboidal Teflon molds were filled with acrylic resin and allowed to cure, thus encasing each specimen while allowing the buccal surface of dentin to be exposed. Each tooth was oriented so that its labial surface was parallel to the shearing force. The teeth were gently dried to expose completely clean enamel. The teeth were randomly assigned into four groups of 60 specimens each:

Group 1—Control: enamel surface was not in contact with acid drinks, acid etching application, and self-adhesive technique for fissure sealant;

Group 2—enamel surface was not in contact with acid drinks, acid etching, and adhesive applications, an etch-and-rinse technique for fissure sealant;

Group 3—enamel surface was immersed for 24 h in acid drink, acid etching application, and self-adhesive technique for fissure sealant;

Group 4—enamel surface was immersed for 24 h in acid drink, acid etching, and adhesive applications, an etch-and-rinse technique for fissure sealant.

Each group was then divided into three subgroups of twenty teeth each according to the fissure sealant used.

Specimens assigned to Groups 3 and 4 were each immersed in 50 mL of a soft drink (Coca-Cola, Coca-Cola Company, Milano, Italy, pH = 2.37) at a temperature of 22 ± 1 °C for 24 h, while specimens assigned to Groups 1 and 2 were immersed in distilled water at a temperature of 22 ± 1 °C for 24 h.

All specimens prepared were etched on a demarcated area of enamel using 37% phosphoric acid for 30 s (Vococid; Voco Gmbh, Cuxhaven, Germany).

Specimens assigned to Groups 2 and 4 were treated with a universal adhesive (Futurabond M+, Voco Gmbh, Cuxhaven, Germany) on the previously etched enamel area. The adhesive was gently air-dried for 5 s and then cured for 10 s using a light-emitting diode (LED) curing light in soft start-polymerization mode (Celalux 2 High-Power LED curing unit; Voco GmbH, Cuxhaven, Germany) at a light intensity of 1000 mW/cm^2^.

### 2.2. Application of Materials Tested

The fissure sealants tested in this study were Fissurit (Voco Gmbh, Cuxhaven, Germany), Grandio Seal (Voco Gmbh, Cuxhaven, Germany), and Admira Fusion (Voco Gmbh, Cuxhaven, Germany). The specifications of the adhesive system used and of all fissure sealants tested are listed in Table 1.

The fissure sealants were applied to the demarcated enamel area using silicon rings (height 2 mm; internal diameter 6 mm; external diameter 8 mm) to obtain specimens identical in size. The cavity of these rings were slightly overfilled with each sealant, covered with a Mylar strip (Henry Schein, Melville, NY, USA), and pressed against a glass plate. All specimens were then light-cured using a LED curing light in soft start-polymerization mode (Celalux 2 High-Power LED curing-light, Voco GmbH, Cuxhaven, Germany) for the times suggested by the manufacturers at an irradiance of 1000 mW/cm^2^. The light was perpendicular to the specimen surface at a distance of 1.5 mm. Following polymerization, specimens were stored in distilled water for 24 h at 37 °C. For each of the four groups described ahead we obtained 20 specimens for each fissure sealant.

### 2.3. Shear Bond Strength Testing

**Figure 1:**
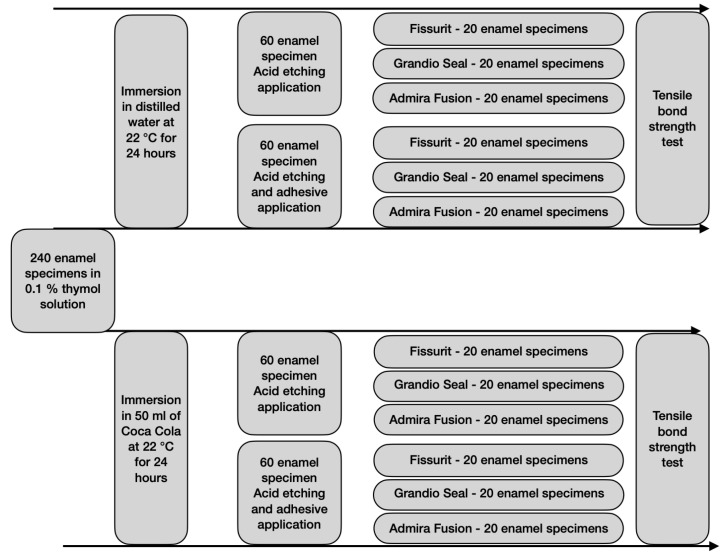
Flow chart of the grouping and testing procedures.

After storing, the specimens were tested in a universal testing machine (Model 3343, Instron Corporation, Norwood, MA, USA). Specimens were secured in the lower jaw of the machine so that the bonded cylinder base was parallel to the shear force direction. The tensile bond strength was performed at 0.5 mm/min until the sample rupture. Specimens were stressed in an occluso-gingival direction at a crosshead speed of 1 mm/min [30,31,32,33,34]. The entire procedure of grouping and testing is represented in Figure 1. The maximum load necessary to debond was recorded in Newton (N) and calculated in MPa as a ratio of Newton to the surface area of the cylinder. After the testing procedure, the fractured surfaces were examined in an optical microscope (Stereomicroscope SR, Zeiss, Oberkochen, Germany) at a magnification of 10x to determine failure modes and classified as adhesive failures, cohesive failures within the composite, or cohesive failures within the tooth [29]. The adhesive remnant index (ARI) was used to assess the amount of adhesive left on the enamel surface [30]. This scale ranges from 0 to 3. A score of 0 indicates no adhesive remaining on the tooth in the bonding area; 1 indicates less than half of the adhesive remaining on the tooth; 2 indicates more than half of the adhesive remaining on the tooth, and 3 indicates all adhesive remaining on the tooth. The ARI scores were used as a method of defining bond failure site among the enamel, the adhesive, and the composite. In our study ARI score is referred to the shear bond strength of the fissure sealants and of adhesive; respectively in groups 1, 3, and groups 2, 4.

### 2.4. Statistical Analysis

Statistical analysis was performed with software (R^®^ version 3.1.3, R Development Core Team, R Foundation for Statistical Computing, Vienna, Austria). Descriptive statistics, including the mean, standard deviation, median, and minimum and maximum values were calculated for all groups. Kolmogorov–Smirnov (KS) tests were applied to assess the normality of distributions. An analysis of variance (two-way ANOVA) was applied to determine whether significant differences in debond values existed among the groups. The Tukey post-hoc test was used. The chi-squared test was used to determine significant differences in the ARI scores among the different groups. Significance for all statistical tests was predetermined at *p* < 0.05.

## 3. Result

**Figure 2:**
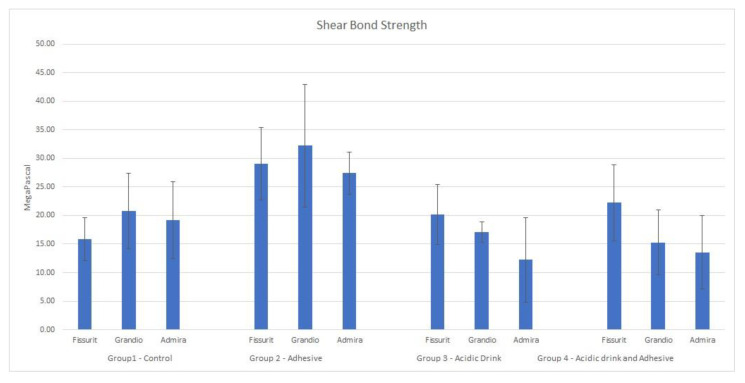
Mean and SD of the different groups tested.

Descriptive statistics of the shear bond strength (MPa) of the different groups are illustrated in Table 2 and Figure 2. Data derived from the analyses were assessed to be normally distributed with the Kolmogorov–Smirnov test (*p* > 0.05). Analysis of variance showed significant differences in shear bond strength among the four groups tested. Post hoc Tukey test evidenced that methodology of Group 2 (no acidic drink applied and etch-and-rinse adhesive system) gave the highest results in terms of MPa for each material tested (*p* < 0.05). Shear bond strength for specimens of Groups 1, 3, and 4 were similar in MPa values (*p* > 0.05). The analysis of adhesive remnant index (ARI) showed that in Groups 2 the adhesive remaining on the bonding area after shear bond strength testing was significantly higher for Admira Fusion and Fissurit where more than half of specimens had ARI = 0. In Group 4 Fissurit maintained similar results, while specimens of Grandio Seal and Admira Fusion detached significantly more for adhesive-sealant binding failure. In Group 1 ARI score was significantly different for each material tested (Table 3). Admira Fusion and Grandio Seal showed similar results and detachment was confined in an enamel-sealant binding failure, while specimens of Fissurit showed a significantly higher frequency of ARI = 3. Differently, in Group 3 Fissurit, Grandio Seal, and Admira Fusion showed a more frequent detachment described by ARI = 0.

## 4. Discussion

The pH of dietary beverages consumed with high frequency appears to be the main cause of enamel erosion in young patients. The enamel softening and consequent dissolution is not directly provoked by the titratable acidity or buffer acidity because of the limited time of contact with enamel surfaces. Therefore, the chemical parameter to focus on to define if a beverage is potentially erosive for dental tissues is the hydrogen ion concentration or pH [35,36,37].

In this study, we tested the influence of Coca-Cola (Coca-Cola Company, Milano, Italy, pH = 2.37), which is globally recognized as an acidic drink and highly consumed by young patients. The in vitro immersion protocol applied to enamel specimens simulates an everyday low-medium consumption prolonged for months. The pH registered for the acidic drink is 2.37, which is underneath the limit of 3, and then it is considered extremely erosive [38,39]. The high concentration of triprotic acids with available hydrogen ions, such as phosphoric acid and citric acid, spurs the dissolution by removing calcium ions from enamel surfaces [40]. For this reason, the apatite solubility and enamel erosion increase logarithmically due to a decreased pH of the oral environment [41].

In the present study, these phenomena of enamel erosion did not cause a significant effect when considering the shear bond strength of different fissure sealants. As reported in Table 1, the test Groups where we developed and applied the immersion protocol to specimens did not show statistically different MPa values for the shear bond strength if compared to the control Group. The application of the adhesive system is not eroded enamel specimens strengthened the binding and MPa values recorded were significantly higher, however, the application of the adhesive did not give any adjunctive bond strength when the enamel was pretreated with the acidic drink. The possible explanation of these results could be the fact that the immersion in acidic drinks modified the ultrastructural morphology of the enamel surface, thus pairing the possible advantages of the adhesive application. As previously described, acidic drinks and free radicals dissolve the enamel surfaces, and the residual glycosaminoglycans components and smear layer on the tooth surface could interfere with proper adhesion with the adhesive system [42,43].

Further SEM analysis should be promoted to magnificate the enamel surfaces treated with acidic drinks and to better understand the reasons for this significant reduction in shear bond strength. The application of the adhesive system seems to be useless when applied on enamel surfaces treated with acidic beverages. This paradigm shift in adhesive techniques could influence not only the procedures of fissure sealants as described in this study but also restorative dentistry and orthodontics. In fact, enamel dissolution is not exclusively a pediatric condition, but, as reported in epidemiological studies, it involves between 16% and 43% of patients of all ages [44]. For orthodontic and prosthodontic purposes, furthermore specific in vitro analysis, such as the tensile bond strength, could be necessary to fully acknowledge the impact of enamel erosion caused by acidic drinks. In the present study, we considered the application of tensile strength on fissure sealants not reliable, and besides this fact, we performed a shear bond strength test.

One of the limitations of this study could be the choice of bovine teeth as a substitute for human teeth. However, this choice allowed us to collect many more samples and thus empower the statistical analysis. Moreover, several authors reported that the chemical structure of bovine and human teeth, even if not identical, does not show significant differences [45,46,47,48]. Another aspect of this research that could be improved is the number of materials tested. Nowadays fissure sealants are widespread and there are several commercial products available for clinicians with a quite different polymerization shrinkage, degree of conversion, and biomechanical properties. These variables related to the composition of the materials could influence the results of the present report [49]. This concern should be evaluated in future studies.

## 5. Conclusions

The tested hypothesis that fissure sealants provide an acceptable shear bond strength when used on eroded enamel with a self-adhesive technique is rejected. When shear bond strength is compared to the values obtained on intact enamel surfaces, a significant difference is assessed and confirmed by statistical analysis. The dissolution of enamel surfaces provokes a reduction in the enamel-adhesive binding due to ultrastructural modifications of the enamel surfaces.

## Figures and Tables

**Table 1:** Adhesive system used for Groups 2–4 and fissure sealants tested.

Material	Type	Composition	Batch Number	Manufacturer
**Futurabond M+**	Universal adhesive	2-Hydroxyethyl methacrylate, BIS-GMA, acidic adhesive monomer, Urethane dimethacrylate, catalyst, pyrogenic silicic acids	1,452,245	Voco, Cuxhaven, Germany
**Fissurit**	Fissure sealant	Matrix: Bis-GMA, diurethane dimethacrylate, BHT, benzotriazolderivate Filler: pyrogenic silicic acid	1,716,509	Voco, Cuxhaven, Germany
**Grandio Seal**	Fissure sealant	Matrix: Bis-GMA, triethylene glycol dymethacrylate (TEGDMA), BHT Filler: pyrogenic silicic acid	1,854,423	Voco, Cuxhaven, Germany
**Admira Fusion**	Fissure sealant	Matrix: Aromatic and aliphatic dimethacrylates, methacrylate-functionalized polysiloxane Filler: Barium-aluminum-glass, pyrogenic silicon dioxide	1,601,001	Voco, Cuxhaven, Germany

**Table 2:** Descriptive statistics (in MPa) of shear bond strengths of the 3 materials tested for each experimental Group. SD: Standard deviation. Different capital letters in the Significance column indicate significant differences in MPa for bond strength (*p* < 0.05).

Groups	Materials	Mean	SD	Min	Mdn	Max	Significance
Group 1—Control	Fissurit	15.83	3.79	13.49	13.79	20.21	A
	Grandio Seal	20.78	6.62	15.00	17.30	29.07	A
	Admira Fusion	19.21	6.68	12.73	18.31	27.48	A
Group 2—Adhesive	Fissurit	29.03	6.34	22.07	28.33	37.39	B
	Grandio Seal	32.26	10.73	23.15	29.26	47.36	B
	Admira Fusion	27.39	3.71	21.99	29.90	30.07	B
Group 3—Acidic drink	Fissurit	20.16	5.27	11.01	22.05	24.26	A
	Grandio Seal	17.10	1.74	15.35	16.78	19.48	A
	Admira Fusion	12.22	7.36	4.10	11.46	21.87	A
Group 4—Acidic drink and adhesive	Fissurit	22.25	6.67	14.15	22.10	32.30	A
	Grandio Seal	15.29	5.63	10.06	14.30	22.49	A
	Admira Fusion	13.56	6.39	9.22	9.43	23.64	A

**Table 3:** Percentages of adhesive remnant index (ARI) for each material tested.

Groups	Materials	ARI = 0	ARI = 1	ARI = 2	ARI = 3
Group 1—Control	Fissurit	20	20	0	60
	Grandio Seal	40	60	0	0
	Admira Fusion	80	20	0	0
Group 2—Adhesive	Fissurit	80	0	0	20
	Grandio Seal	20	80	0	0
	Admira Fusion	100	0	0	0
Group 3—Acidic drink	Fissurit	60	20	20	0
	Grandio Seal	60	20	0	20
	Admira Fusion	60	0	20	20
Group 4—Acidic drink and adhesive	Fissurit	80	20	0	0
	Grandio Seal	40	0	0	60
	Admira Fusion	40	0	0	60

0 = no sealant remaining on the tooth in the bonding area. 1 = less than half of the sealant remaining on the tooth, 2 = more than half of the sealant remaining on the tooth, 3 = all sealant remaining on the tooth.

## Data Availability

All data are available upon request to Corresponding Author.
